# Comparative Effectiveness of Midazolam-Based Sedation on the Need for Intracranial Pressure Lowering Therapies in Traumatic Brain Injury

**DOI:** 10.1089/neur.2024.0077

**Published:** 2025-03-05

**Authors:** Rianne G.F. Dolmans, Giovanni Russo, James Anstey, Ewout W. Steyerberg, Fabio S. Taccone, Andrew Udy, Giuseppe Citerio, Carole Ichai, Rafael Badenes, John Prowle, Ari Ercole, Mauro Oddo, Antoine Schneider, Stefan Wolf, Raimund Helbok, David Nelson, D. Jamie Cooper, Mathieu van der Jagt

**Affiliations:** ^1^ Department of Neurosurgery, Leiden University Medical Center, Leiden, The Netherlands.; ^2^ Intensive Care Unit, Royal Melbourne Hospital, Melbourne, Victoria, Australia.; ^3^ Department of Biomedical Data Sciences, Leiden University Medical Center, Leiden, The Netherlands.; ^4^ Department of Intensive Care, Hôpital Universitaire de Bruxelles, Université Libre de Bruxelles, Brussels, Belgium.; ^5^ Intensive Care Unit, Alfred Hospital, Melbourne, Victoria, Australia.; ^6^ Australian and New Zealand Intensive Care Research Centre, School of Public Health and Preventive Medicine, Monash University, Melbourne, Victoria, Australia.; ^7^ School of Medicine and Surgery, Fondazione IRCCS San Gerardo dei Tintori, University Milano Bicocca - Neurointensive Care, Monza, Italy.; ^8^ Center Hospitalier Universitaire de Nice, Service de Réanimation polyvalente, Université Côte d’Azur, Nice, France.; ^9^ Department of Anesthesiology and Surgical-Trauma Intensive Care, Hospital Clinic Universitari de Valencia, University of Valencia, Valencia, Spain.; ^10^ Adult Critical Care Unit, Royal London Hospital, Barts Health NHS Trust, London, United Kingdom.; ^11^ Neurosciences and Trauma Critical Care Unit, Cambridge University Hospitals NHS Foundation Trust, Cambridge, United Kingdom.; ^12^ Direction of Innovation and Clinical Research, Centre Hospitalier Universitaire Vaudois (CHUV) Direction, Faculty of Biology and Medicine, University of Lausanne, Lausanne, Switzerland.; ^13^ Department of Medical-Surgical Intensive Care Medicine, Faculty of Biology and Medicine, Center Hospitalier Universitaire, Vaudois (CHUV), University of Lausanne, Lausanne, Switzerland.; ^14^ Department of Neurosurgery, Charité Universitätsmedizin Neuro Intensive Care Unit, Berlin, Germany.; ^15^ Department of Neurology, Neurocritical Care Unit, Medical University of Innsbruck, Innsbruck, Austria.; ^16^ Department of Neurology, Kepler University Hospital, Johannes Kepler University Linz, Linz, Austria.; ^17^ Function Perioperative Medicine and Intensive Care, Karolinska University Hospital, Stockholm, Sweden.; ^18^ Department of Intensive Care Adults, Erasmus Medical Center, Rotterdam, The Netherlands.

**Keywords:** intracranial pressure, neurocritical care, sedation, traumatic brain injury

## Abstract

Sedatives play an important role in the management of patients with severe traumatic brain injury (sTBI) in the intensive care unit (ICU). Benzodiazepines are common for sedation (midazolam-based) but have been discouraged for non-brain-injured patients in the ICU. This study aimed to investigate the effect of midazolam-based sedation versus non-midazolam-based sedation on the need for intracranial pressure (ICP) lowering therapies in patients with sTBI in the ICU. We studied patients with sTBI (Glasgow Coma Sale ≤8) from 14 ICUs in Europe and Australia, who received ICP monitoring and continuous instrumental variable (IV) sedation for at least 24 h. We analyzed the association between sedation strategy and the need for ICP lowering therapies during the first 7 ICU days using a multivariable logistic regression model, adjusted for clinical markers of injury severity. We also analyzed the center as an IV in a random effects model to address potentially unmeasured confounding. Among 227 patients with sTBI, 152 (67%) received midazolam-based sedation. These patients had a lower age and higher median Glasgow Coma Scale on admission compared with 75 patients in the non-midazolam-sedated group. In logistic regression analyses, patients with midazolam-based sedation had higher odds of receiving hyperosmolar therapy (odds ratio [OR]: 3.4, 95% confidence intervals [CI]: 1.6–7.7). This effect could not be confirmed in the instrumental variable analysis (hyperosmolar therapy: OR: 1.3, 95% CI: 0.1–13.1). The mean ICU length of stay was significantly longer in the midazolam-based sedation group compared with the non-midazolam-based sedation group (19 vs. 13 days, hazards ratio 0.6, 95% CI: 0.4–0.8). Midazolam-based sedation was common for patients with sTBI without a significantly increased need for ICP therapies but an association with longer ICU stay. Larger prospective comparative effectiveness studies are needed regarding sedation strategies in critically ill patients with TBI.

## Introduction 

Sedatives and analgesics play an important role in the management of patients with severe traumatic brain injury (sTBI) in the intensive care unit (ICU), which is aimed at the prevention of secondary brain injury (SBI).^1–4^ There is wide variation, however, in the use of sedative agents because of a lack of evidence and guidelines on their use.^2,5,6^ Benzodiazepines have been used for sedation in patients with TBI for many years in the ICU setting, with midazolam being the most common.^4^ Midazolam has the advantage of less severe side effects (e.g., hemodynamic instability) compared with most other sedative agents, and therefore, it could be intensified more easily when the therapy intensity level (TIL) is escalated.^4,7–10^ Disadvantages of midazolam may include tissue accumulation, especially in patients with renal dysfunction, with prolonged sedation and duration of mechanical ventilation.^4,7,11–13^ Besides midazolam, there is increased use of propofol and other sedative agents such as dexmedetomidine and clonidine.^11^ Some appear to be equally effective in managing intracranial pressure (ICP), one of the main modifiable components of SBI, but with less tissue accumulation and shorter drug-elimination time.^4,11^

A previous study investigated the early use (first 24–72 h after ICU admission) of sedative agents in relation to clinical outcomes in patients with sTBI in the ICU.^14^ The current study aimed to investigate the association of midazolam-based sedation versus non-midazolam-based sedation with the need for ICP lowering therapies during the first 7 days in patients with sTBI in the ICU.

## Materials and Methods

### Study setting and data collection

In this study, data from 14 ICUs in Europe and Australia were prospectively collected and analyzed. Two centers were from Melbourne, Australia, 2 were from the United Kingdom (London and Cambridge), and the other 10 were from Europe (Valencia, Spain, Innsbruck, Austria, Stockholm, Sweden, Lausanne, Switzerland, Monza, Italy, Paris and Nice, France, Berlin, Germany, Brussels, Belgium, Rotterdam, Netherlands). Ethics approval for this study was obtained locally by each center. Centers collected data from at least 20 patients each. Inclusion criteria were adult patients (>18 years of age) with sTBI (i.e., Glasgow Coma Scale [GCS] ≤8 after resuscitation or prior to intubation). All patients received ICP monitoring by an external ventricular drain (EVD) or intraparenchymal catheter within 24 h of admission that remained *in situ* for at least 72 h of their ICU stay. In addition, all patients received continuous intravenous sedation with midazolam and/or propofol and/or dexmedetomidine and/or clonidine for at least 24 h. Exclusion criteria were death within <72 h from admission as well as rapid improvement within 72 h, permitting removal of their ICP monitor before this time. Baseline characteristics, vital signs, injury severity scores ([ISS], GCS, pupil reactivity, Marshall computed tomography [CT] score, APACHE II, IMPACT score), sedation data, and ICP lowering therapies were collected for each patient using the clinical records of the patients ICU stay. Sedation infusion rates were recorded at midday each day. ICP was assessed four times a day. Clinical outcomes such as ICU length of stay, hospital length of stay, ICU mortality, in-hospital mortality, and Glasgow Outcome Scale Extended (GOS-E) at hospital discharge have already been reported in a previous study.^14^ We report these outcomes for completeness because analyses and patient selection for this study were slightly different. GOS-E at hospital discharge was defined as deceased (GOS-E 1), unfavorable outcome (GOS-E 2–4), or favorable outcome (GOS-E 5–8).

### Outcome measurements

The primary outcome measurements were ICP lowering therapies at any time during ICU stay as defined in the TIL score that was available from the data collected and included EVD placement for cerebrospinal fluid (CSF) drainage, barbiturate coma, neurosurgical clot evacuation, decompressive craniectomy, therapeutic hypothermia (temperature 32–35°C), and use of hyperosmolar therapy (mannitol and/or hypertonic saline). A summary TIL score was not possible due to a lack of dosing data. ICU and hospital length of stay were secondary outcomes.

### Statistical analysis

Patients were stratified into two groups: midazolam-based sedation versus non-midazolam-based sedation. We defined the “midazolam-based group” as those patients treated with continuous intravenous midazolam infusion alone or with another sedative agent for 1 or more days during the first 7 days of their ICU stay. The “non-midazolam-based group” were patients treated with propofol and/or clonidine and/or dexmedetomidine or a combination of these (as long as they did not receive midazolam) during the first 7 days of their ICU stay. The chi-square exact test was used to compare categorical variables, and the Kruskal–Wallis test was used to compare continuous variables. Multivariable logistic regression models, adjusted for clinical markers of injury severity (ISS, GCS, pupil reactivity, Marshall CT score, APACHE II, IMPACT score), were used to analyze the association between sedation strategy and any ICP lowering therapy as well as the six individual, dichotomized (yes/no) ICP lowering therapies as outcomes. Moreover, an instrumental variable analysis was performed with a preference for midazolam per center as the exposure variable. Preference for midazolam per center was defined as the chance of getting midazolam-based sedation at each center. A random effect model adjusted for unmeasured confounding based on the center effect. Finally, a sensitivity analysis was performed excluding patients with a primary decompressive craniectomy on day one. For secondary outcome analyses, a Cox proportional hazards model was used. All statistical analyses were performed in R Studio (version 2022.07.1). A *p* value below 0.05 was considered significant.

## Results

### Baseline characteristics

**FIG. 1.**
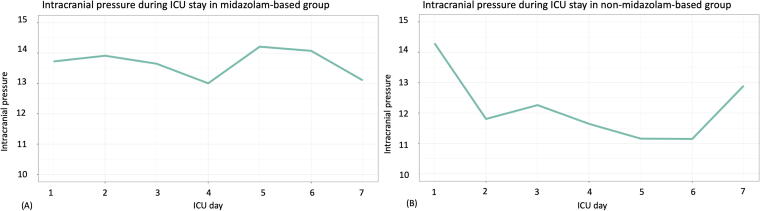
Mean intracranial pressure during ICU stay in patients with midazolam-based **(A)** and non-midazolam-based sedation **(B)**. ICU, intensive care unit.

In total, 227 patients with sTBI were included, of whom 152 patients (67%) received midazolam-based sedation and 75 patients (33%) received non-midazolam-based sedation. Mean age was lower in the midazolam-based sedation group compared with the non-midazolam-based sedation group (43 ± 19 years vs. 49 ± 18 years). Patients in the midazolam-based group had a higher median GCS total score on admission (3, interquartile range [IQR]: 3–8 vs. 3, IQR: 3–5) as well as higher median GCS motor score on admission (1, IQR: 1–5 vs. 1, IQR: 1–2) compared with patients in the non-midazolam-based sedation group. Prehospital hypoxemia (25% vs. 10%, respectively), as well as opioid use (100% vs. 89%, respectively), was more common in the midazolam-based group compared with the non-midazolam-based group (Table 1). The IMPACT score was lower in the midazolam group (10, IQR: 7–12) versus the non-midazolam group (12, IQR: 9–14). The mean ICP course between the midazolam-based sedation group and the non-midazolam-based sedation group is shown in Figure 1.

**Table 1.** Baseline Demographics

	Midazolam-based sedation	Non-midazolam-based sedation
Number of patients	152 (67)	75 (33)
Age (mean [SD])	42.5 (±19)	49.1 (±18)
Gender (%)		
Female	31 (20)	18 (24)
Male	121 (80)	57 (76)
Admission weight (mean [SD])	77 (14)	88 (107)
APACHE II score (median [IQR])	19 (15, 25)	21 (16, 26)
IMPACT score (median [IQR])	10 (7, 12)	12 (9, 14)
Injury Severity Score (median [IQR])	32 (25, 42)	29 (25, 38)
GCS on admission (median [IQR])	3 (3, 8)	3 (3, 5)
GCS motor score on admission (median [IQR])	1 (1, 5)	1 (1, 2)
Prehospital hypoxemia (SpO_2_ < 90%; %)	34 (25)	7 (10)
Prehospital hypotension (SBP <90 mmHg; %)	24 (17)	12 (17)
Prehospital fluid volume given (mean [SD])	887 (907)	767 (1131)
Prehospital osmotherapy (%)	12 (10)	8 (20)
Pupil reactivity (%)		
Non-reactive	23 (16)	14 (19)
One reactive	18 (12)	15 (20)
Both reactive	104 (72)	45 (61)
Mass lesion on CT (%)	113 (72)	41 (59)
Traumatic subarachnoid hemorrhage (%)	109 (72)	53 (71)
Marshall CT score (median [IQR])	3 (2, 5)	3 (2, 5)
Mean ICP (SD)	14 (4)	12 (3)
Opioid use	152 (100)	67 (89)
NMBA use	42 (28)	14 (19)

CT, computed tomography; GCS, Glasgow Coma Scale; ICP, intracranial pressure; IQR, interquartile range; NMBA, neuromuscular blockade agents; SBP, systolic blood pressure; SD, standard deviation.

### ICP lowering therapies

ICP lowering therapies were given in 84% of patients with midazolam-based sedation and in 72% of patients with non-midazolam-based sedation. Specifically, additional ICP lowering therapies such as therapeutic hypothermia (32% vs. 17%), barbiturate coma (16% vs. 5%), and hyperosmolar therapy (68% vs. 41%) were more common in the 152 patients in the midazolam-based sedation group compared with the 75 patients in the non-midazolam-based sedation group, respectively. Other ICP lowering therapies such as neurosurgery for clot evacuation, craniectomy, and EVD requirement for CSF drainage were not different between groups (Table 2).

**Table 2.** ICP Lowering Therapies by Sedation Strategy

	Midazolam-based sedation	Non-midazolam-based sedation
Number of patients	152	75
Any ICP lowering therapies	128 (84)	54 (72)
Therapeutic hypothermia (%)	48 (32)	13 (17)
Barbiturate coma (%)	24 (16)	4 (5)
Neurosurgery for clot evacuation (%)	50 (35)	24 (35)
Craniectomy (%)	48 (32)	19 (25)
Craniectomy on day 1 (%)	46 (30)	19 (25)
EVD required for CSF drainage (%)	53 (35)	16 (21)
Hyperosmolar therapy	103 (68)	31 (41)

CSF, cerebrospinal fluid; EVD, external ventricular drain; ICP, intracranial pressure.

### Sedation strategy and ICP lowering therapies

On multivariable logistic regression analysis, when correcting for multiple potential confounders (e.g., age, gender, admission weight, injury severity score, IMPACT score, and opioid use), we found that patients with midazolam-based sedation had higher odds of receiving hyperosmolar therapy (odds ratio [OR]: 3.4, 95% confidence interval [CI]: 1.6–7.7) compared with patients with non-midazolam-based sedation. The odds of any ICP lowering therapy or therapeutic hypothermia, EVD for CSF drainage, neurosurgery for clot evacuation, and craniectomy were comparable between groups. The number of patients with barbiturate coma was low (16% in midazolam groups vs. 5% in non-midazolam group), and all patients with barbiturate coma received opioids. Therefore, we were unable to perform a regression analysis with correction for confounders for this specific ICP therapy (Table 3). In the instrumental variable model, there was no statistically significant association between midazolam preference versus non-midazolam preference and the need for ICP lowering therapies (Table 3).

**Table 3.** Multivariable Logistic Regression and Random Effects Model on Midazolam-Based Sedation Versus Non-Midazolam-Based Sedation in Relation to the Need for ICP Lowering Therapies

	Any ICP lowering therapy	Therapeutic hypothermia	Barbiturate coma	Neurosurgery for clot evacuation	Craniectomy	EVD required for CSF drainage	Hyper-osmolar therapy
	OR (95% CI)	OR (95% CI)	OR (95% CI)	OR (95% CI)	OR (95% CI)	OR (95% CI)	OR (95% CI)
Multivariate logistic regression model^a^							
Midazolam-based sedation	1.9 (0.7–4.6)	1.9 (0.8–4.9)	NA	0.9 (0.4–2.0)	1.2 (0.5–3.0)	1.8 (0.8–4.8)	**3.4 (1.6–7.7)**
Non-midazolam-based sedation	Ref	Ref	NA	Ref	Ref	Ref	Ref
Random effects model^a^							
Center with midazolam preference versus non-midazolam preference	2.2 (0.1–42.2)	12.3 (0.9–164)	2.8 (0.1–164)	0.7 (0.1–4.9)	0.5 (0.1–3.5)	12.5 (0.3–483)	0.9 (0.1–7.9)
Center with midazolam preference versus non-midazolam preference—corrected for covariates	3.7 (0.1–125)	7.3 (0.3–167)	NA	0.6 (0.1–8.0)	0.5 (0.02–9.8)	21 (0.1–4285)	1.3 (0.1–13.1)
Random effects model: Calculated per 10% more preference for treatment^a^							
Midazolam preference	1.1 (0.8–1.5)	1.3 (1.0–1.7)	1.1 (0.7–1.7)	1.0 (0.8–1.2)	0.9 (0.8–1.1)	1.3 (0.9–1.9)	1.0 (0.8–1.2)
Midazolam preference—corrected for covariates	1.1 (0.7–1.8)	1.2 (0.9–1.7)	1.6 (0.9–3.1)	1.0 (0.7–1.2)	0.9 (0.7–1.3)	1.4 (0.8–2.3)	1.0 (0.8–1.3)

Bold type indicates significance.

^a^ The multivariable logistic regression model and multivariate random effects model were adjusted for age, gender, admission weight, injury severity score, IMPACT score, and opioid use.

CI, confidence interval; CSF, cerebrospinal fluid; EVD, external ventricular drain; ICP, intracranial pressure; OD, odds ratio.

### Sensitivity analysis—craniectomy on day 1

In a sensitivity analysis excluding patients with a primary decompressive craniectomy on day 1, we found similar results where patients with midazolam-based sedation had higher odds of receiving hyperosmolar therapy (OR: 3.8, 95% CI: 1.5–10) as an ICP therapy compared with patients with non-midazolam-based sedation. The odds for the other ICP therapies were not different between groups. In the random effect model, however, we found no significant differences (Table 4).

**Table 4.** Sensitivity Analysis of Sedation in Patients Without Craniectomy on Day 1 in Relation to the Need for ICP Lowering Therapies

	Any ICP lowering therapy	Therapeutic hypothermia	Barbiturate coma	Neurosurgery for clot evacuation	Craniectomy	EVD required for CSF drainage	Hyper-osmolar therapy
	OR (95% CI)	OR (95% CI)	OR (95% CI)	OR (95% CI)	OR (95% CI)	OR (95% CI)	OR (95% CI)
Multivariate logistic regression model^a^							
Midazolam -based sedation	2.0 (0.7–5.2)	1.8 (0.7–5.4)	NA	1.3 (0.5–4.1)	NA	1.56 (0.6–4.8)	**3.8 (1.5–10.2)**
Non-midazolam-based sedation	Ref	Ref	Ref	Ref	Ref	Ref	Ref
Random effects model^a^							
Center with midazolam preference versus non-midazolam preference—corrected for covariates	6.2 (0.2–165)	4.7 (0.2–146)	NA	1.4 (0.2–11.9)	NA	NA	2.9 (0.3–30.3)
Random effects model: Calculated per 10% more preference for treatment^a^							
Midazolam preference—corrected for covariates	1.2 (0.9–1.7)	1.2 (0.8–1.7)	1.7 (0.8–3.5)	1.0 (0.8–1.3)	NA	1.4 (0.6–3.0)	1.1 (0.9–1.4)

^a^ The multivariable logistic regression model and multivariate random effects model were adjusted for: age, gender, admission weight, injury severity score, IMPACT score, and opioid use.

CI, confidence interval; CSF, cerebrospinal fluid; EVD, external ventricular drain; ICP, intracranial pressure; OD, odds ratio.Bold type indicates significance.

### Clinical outcomes

Hospital length of stay, ICU mortality, in-hospital mortality, and GOS-E at hospital discharge were similar in both groups. ICU length of stay, however, was significantly longer in the midazolam-based sedation group compared with the non-midazolam-based sedation group (19 ± 15 days vs. 13 ± 8 days, respectively; Table 5). When adjusting for confounding factors, these results remained statistically significant (hazards ratio 0.6, 95% CI: 0.4–0.8; Table 6).

**Table 5.** Clinical Outcomes by Sedation Strategy

	Midazolam-based sedation	Non-midazolam-based sedation
Number of patients	152	75
ICU length of stay (mean [SE])	19 (1)	13 (1)
Hospital length of stay (mean [SE])	32 (3)	24 (2)
ICU mortality (%)	37 (24)	16 (21)
In-hospital mortality (%)	41 (27)	18 (24)
Glasgow outcome scale extended at hospital discharge (%)		
Deceased	41 (37)	18 (32)
Favorable	14 (13)	9 (16)
Unfavorable	56 (51)	29 (52)

ICU, intensive care unit; SE, standard error.

**Table 6.** Cox Regression Model on ICU and Hospital Length of Stay

	ICU length of stay	Hospital length of stay
	HR (95% CI)	HR (95% CI)
Midazolam-based sedation	**0.6 (0.4–0.8)**	1.1 (0.8–1.6)
Non-midazolam-based sedation	Ref	Ref

The statistical model was adjusted for age, gender, admission weight, injury severity score, IMPACT score, and opioid use.

CI, confidence interval; HR, hazards ratio; ICU, intensive care unit.Bold type indicates significance.

## Discussion

We found that most patients with sTBI had midazolam-based sedation. These patients received somewhat more treatment escalations (hyperosmolar therapy, therapeutic hypothermia, EVD for CSF drainage, neurosurgery for clot evacuation, and craniectomy as ICP lowering therapies) compared with patients with non-midazolam-based sedation. Differences were not confirmed, however, in instrumental variable analyses, which adjusted for center-level preference as the instrument. This suggests that using midazolam was not superior in controlling ICP, compared with other drugs, in this patient cohort. Furthermore, ICU length of stay was longer when using midazolam-based sedation, which aligns with previous research in non-TBI populations.^15,16^ This comparative effectiveness study focused on ICP treatments. A previous report from the same databases showed similar clinical outcomes with both sedation strategies.^14^ Hence, both clinical outcomes and TIL for ICP appear relatively independent of using midazolam as a component of the sedation strategy.

Previous research has been performed on the influence of sedative choice on cerebral (ICP, cerebral perfusion pressure (CPP), cerebral blood flow (CBF), cerebral metabolic rate of oxygen (CMRO_2_) and systemic (mean arterial pressure; MAP) physiology parameters.^3,6,17–37^ Even though randomized controlled trials are scarce,^20,36–38^ and the majority of research are small interventional trials or observational studies, no clear relation has been found between sedative choice and cerebral and systemic physiology parameters to date. This results in a lack of consensus on the preferred method of sedation in patients with TBI in the ICU.^2^ To the best of our knowledge, this is the first study comparing sedation in relation to the need for ICP lowering therapies.

The current study investigated six ICP lowering therapies: therapeutic hypothermia, barbiturate coma, neurosurgery for clot evacuation, EVD for CSF drainage, craniectomy, and hyperosmolar therapy. Comparing midazolam versus other sedative agents in patients with TBI has been the topic of many studies. To date, none of these studies, however, have found superiority of midazolam over other sedative agents or vice versa in terms of controlling ICP,^21,39^ cerebral oxidative stress,^31^ markers of neurological injury,^40^ or neurological outcome.^21,39,40^ Nevertheless, propofol is recommended for the control of ICP,^41^ and there is a common clinical assumption that propofol leads to better control of ICP compared with midazolam despite a lack of good quality comparative data.^4^ Our study does not clearly indicate that there may be a clear superiority of sedative agents in terms of ICP lowering therapies in patients with TBI. Nonetheless, the role of sedation on ICP control remains to be discussed since sedatives can be indicated to control other parameters influencing ICP such as arterial hypotension, hypo- or hypercapnia, hypoxia, and increased cerebral oxygen use (CMRO_2_).^42,43^

Primary decompressive craniectomy, removing the bone flap without its replacement for the treatment of intracerebral mass, is commonly performed in patients with TBI to prevent increased ICP.^44–46^ For our sensitivity analysis, we have excluded patients undergoing a primary decompressive craniectomy on day 1 with the assumption that they will need fewer ICP therapies since ICP may immediately be controlled. Therefore, this group of patients might have skewed the results of our analysis. Nevertheless, the sensitivity analysis indicated similar results.

Regarding sedative choice and its relation with clinical outcomes, the current study is in line with previous research indicating that patients with TBI with midazolam-based sedation have longer ICU length of stay.^3,9,21,47^ This may be related to the accumulation of active metabolites with prolonged infusions,^48^ with no differences in mortality or GOS-E at hospital discharge.^39,40^

A limitation of the current study is the retrospective nature of this research, with a substantial number of analyses done. We caution that the substantial number of analyses increases the chance of focusing on a chance finding rather than a true causal relationship. Moreover, long-term neurological outcomes as well as depth of sedation were not collected. Nor were we able to investigate renal function in the midazolam-based sedation group which can be a confounder for ICU length of stay due to accumulation of active metabolites in renal failure.^49^ We could not include the validated TIL score due to insufficient granular data in this dataset. Instead, we included most components of the TIL score as binary variables, when available. One of the strengths of this article is that the statistical analyses included various state-of-the-art approaches, including instrumental variable analyses. Also, this is an international multicenter study including centers from both Europe and Australia, increasing generalizability due to a diverse population coverage. In addition, confounder adjustment included instrumental variable analysis with the center effect, which theoretically leverages variability between centers that is not accounted for by confounding by indication, enhancing the potential of the associations found to be interpreted as being causal in relation to propensity scores.^50^ The disadvantage of this refined analysis is a large variance due to low statistical power. The number of patients may have been too small to identify the true effects of midazolam-based versus non-midazolam-based sedation if they did exist resulting in no convincing conclusions. More reliable results are only possible with far larger numbers of patients.

## Conclusions

Midazolam-based sedation is common for patients with sTBI and is not clearly related to the need for ICP lowering therapies in patients with sTBI in the ICU compared with non-midazolam-based sedation strategies. Midazolam-based sedation was associated with increased ICU length of stay. More extensive prospective comparative effectiveness studies regarding the benefits of different sedation strategies in critically ill patients with TBI are needed.

## Transparency, Rigor, and Reproducibility Statement

This study was not formally registered because of the retrospective nature of this article. The analysis plan was not formally pre-registered, but the team member with primary responsibility for the analysis certifies that the analysis plan was prespecified. A sample size of 260 subjects was planned based on the availability of the data from the participating centers. The actual sample size was 227 subjects. Data were acquired between January 1, 2015, and December 31, 2015. Data were collected using the clinical records of the patients ICU stay. Data were analyzed using R Studio (version 2022.07.1). All equipment and software used to perform analysis are widely available from R Studio. Key inclusion criteria and clinical outcomes were assessed by investigators with a clinical background in intensive care medicine, neurology, and neurosurgery. Effect sizes and confidence intervals have been reported in the abstract and main text for all outcomes. Statistical analysis and/or review was performed by a qualified statistician (E.W.S.). Methods of statistical analysis have been reported in the text. Data from the study are not available because data cannot be de-identified. The analytic code used to conduct the analyses presented in this study is not available in a public repository. They may be available by emailing the corresponding author. The authors agree or have agreed to publish the article using the Mary Ann Liebert Inc. “Open Access” option under the appropriate license.

## Abbreviations Used

CT: computed tomography
CSF: cerebrospinal fluid
EVD: external ventricular drain
GCS: Glasgow Coma Scale
ICP: intracranial pressure
ICU: intensive care unit
NMBA: neuromuscular blockade agents
SBP: systolic blood pressure
SD: standard deviation
